# Satellite Observation of Spatio-temporal Variations in Nitrogen Dioxide over West Africa and Implications for Regional Air Quality

**DOI:** 10.5696/2156-9614-11.31.210913

**Published:** 2021-08-17

**Authors:** Ayodeji Oluleye

**Affiliations:** Department of Meteorology and Climate Science, Federal University of Technology, Akure, Nigeria

**Keywords:** atmospheric pollution, climate change, nitrogen dioxide, West Africa, bush burning

## Abstract

**Background.:**

Nitrogen dioxide (NO_2_) is known to affect human health, causing heart and cardiovascular diseases, and it has been shown that locations with long term NO_2_ pollution recorded a high number of fatalities due to the COVID-19 pandemic. There are no ground stations monitoring emissions of NO_2_ over West Africa. The present study aimed to use satellite observations to examine pollutant trends over this region.

**Objective.:**

To examine the trend of NO_2_ over the entire West Africa sub region in relationship to contributions to environmental emissions using satellite-derived data. This enables the assessment of West Africa regional air pollution hot spots in relationship to enhancing atmospheric factors. The results from this study will also be useful guidance for setting air quality standards for air pollution controls to minimize health hazards.

**Methods.:**

The present study examined thirteen years of average monthly values of nitrogen dioxide (NO_2_) to determine the spatio-temporal variation of this pollutant over West Africa. Satellite data for NO_2_ between 2005 and 2017 were used to determine the variation in pollution levels over West Africa. Correlations between NO_2_ and meteorological variables (wind speed, rainfall and air temperature) were obtained to explain the influence of West African weather on the region's pollution accumulation.

**Results.:**

The present study observed that NO_2_ concentrations varied from place to place and from season to season. Nitrogen dioxide concentrations during the dry season were higher (sometimes 200% higher) than values observed in the wet season which ranged between 0.5 and 6×10^15^ molec/cm^2^. Nitrogen dioxide north-south oscillation during the course of a year is largely controlled by the inter-tropical discontinuity (ITD) zone as high concentrations of NO_2_ are found in the vicinity of the ITD where wind speeds and horizontal vorticity approaches zero. Correlation analysis between NO_2_ and some atmospheric variables indicated NO_2_ concentrations are well influenced by atmospheric variables showing bipolar signals depending on the season. An increasing trend of NO_2_ was also found over selected cities of the region. This indicated that regional air quality is gradually deteriorating.

**Conclusions.:**

The implications of worsening regional air quality were examined in the light of the prevailing COVID-19 pandemic. The dominant atmospheric factor determining pollution episodes in the region is the inter-tropical discontinuity line which marks the meeting point between the two wind regimes over the region. Densely populated areas are characteristically prone to elevated pollution and have experienced high fatalities during the COVID-19 pandemic.

**Competing Interests.:**

The authors declare no competing financial interests.

## Introduction

Certain atmospheric gases are commonly referred to as criteria pollutants. The amount of the gases present in the atmosphere measured against a set of standard values determines the air quality of a location. Nitrogen dioxide (NO_2_) is a criteria pollutant that has several sources of emissions over West Africa. Both natural and anthropogenic emission sources of NO_2_ are largely uncontrolled over the region. Agricultural activities such as dry biomass from crops and bush burning are widely practiced by farmers who are largely subsistence farmers and lack the technology to control emissions. Fossil fuel burning in industries and vehicular emissions are other unregulated sources of NO_2_ in urban areas around the region. Nitrogen dioxide is also emitted naturally from soils due to bacteria activities and application of fertilizers.^1^

Similarly, a significant amount of NO_2_ is emitted as a result of lightning. However, Peel *et al* has shown that the annual amount of NO_2_ due to anthropogenic sources far exceeds natural emissions.^2^ Nitrogen dioxide is very reactive in the atmosphere, occurring initially as nitrogen monoxide (NO).^3^ Oxidation converts NO to NO_2_.^4^ The conversion process is reversed as a result of photolytic reaction when NO_2_ changes to NO in the presence of sunlight as a catalyst.^5^,^6^ The oxidation–photolysis process continues until equilibrium is reached according to the chemical process represented in Equation 1.


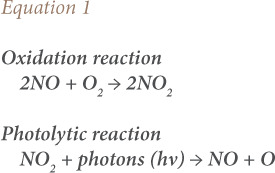


Nitrogen dioxide, whose presence in the atmosphere causes horizontal visibility reduction, is quite active in the production of tropospheric ozone leading to a smog problem in the urban atmospher.^6^ This occurs early in the morning when vehicle emissions are trapped and mixed with fog close to the ground surface due to shallow boundary layer depth.^7^ Nitrogen dioxide dissolves in rainwater through a chemical process to form nitrous acid and nitric acid causing damage to roofing sheets and sculptures.^7^ The presence of NO_2_ in the atmosphere has been shown to constitute a significant human health hazard. It is a leading cause of lung and heart diseases.^8^ Elevated levels of NO_2_ also causes suppression of plant growth and impairs their ability to withstand drought, leading to crop failure and food security crises.^9^

Ground station measurement of NO_2_ is virtually non-existent in West Africa except for some isolated but unreported case studies in the Nigerian Niger Delta. Documented studies of human health impacts and source apportionment of NO_2_ are sparse.^10^ Satellite remote sensing has proved effective in the aerial study of pollution. It offers the advantage of continuous measurement over a large area. An investigation conducted by Marais *et al.* using satellite data to map the concentration of anthropogenic emissions in Nigeria revealed that seasonal variation of NO_2_ matched the occurrence of large open fires over the Niger delta.^11^ These large open fire sources are illegal refineries destroyed by burning, fire from continuous gas flaring and fires from burst pipes, which are criminal acts in most cases. Relevant research studies on West African regional air quality are scarce due to lack of pollution monitoring points and therefore regional air quality is poorly documented and not well understood. The focus of the present study was to examine the trend of NO_2_ over the entire West Africa sub region in relationship to contributions to environmental emissions using satellite-derived data. This enables the assessment of West Africa regional air pollution hot spots in relationship to enhancing atmospheric factors. The results from this study will also be useful guidance for setting air quality standards for air pollution controls to minimize health hazards.

**Abbreviations**
*ECMWF*: European Centre for Medium-range Weather Forecast
*ITD*: Inter-tropical discontinuity
*NLIN*: Non-linear

### Study area meteorology and demography

**Figure 1:**
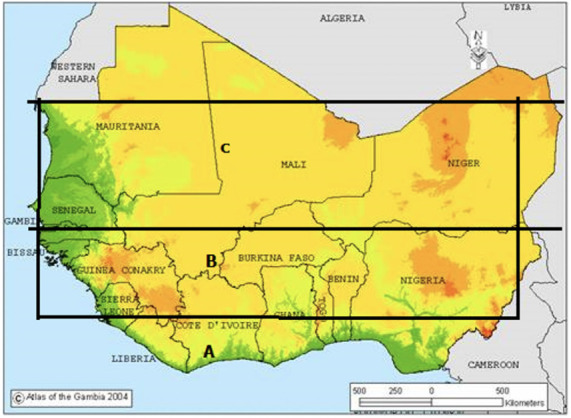
Map of West Africa showing elevation over the region. Bold letters A, B and C show the approximate vegetation demarcation of the Guinea Coast, Savanna and Sahel respectively. Adapted from Atlas of the Gambia^7^

West Africa is bounded by the Gulf of Guinea to the south, the Atlantic Ocean to the west, and the Sahara Desert to the north *(Figure 1).* Weather over the region is controlled by a number of atmospheric factors; prominent among them are the inter-tropical discontinuity (ITD), Africa, Easterly Jet and the monsoon winds and moisture depth within the atmospheric boundary layer.^7^ The monsoon winds have two regimes; south-westerly from the ocean and north-easterly winds from the Sahara Desert. These are two contrasting winds whose directions of flow are opposite and their meeting point at the surface is commonly referred to as the ITD. Rains bearing active weather zones are located about 400 or 500 km south of the ITD. Annual migration of the ITD is sun synchronous.^12^ Thus, four periods could be attributed to the migration of the ITD. In the months of February, March and April, a pre-monsoon period is established when the ITD migrates from its southernmost position on an annual journey to the north. The months of May, June and July mark the period when the monsoon is established from the south up to latitude 10°N. However, in August, the ITD reaches its northernmost position creating a dry season zone over the south. Between September, October and November, the ITD retreats at a faster rate back to the south producing a second rainfall maximum over the south and finally, in December and January, and sometimes extending to March over the north, a period of Harmattan is established when the dry and dusty north-easterly winds predominantly cover the entire region.

Pre-monsoon and Harmattan periods are the time of extensive burning of bush and crop residues in preparation for the early planting season. West Africa is broadly partitioned into three vegetation belts according to Odekunle *et al*. as forest (coast–8 °N), savanna woodland (8°–11 °N) and savanna grassland zones (11°–16 °N).^13^ The forest zone receives more rain than other zones because rainfall decreases from south to north over the region, therefore the woodland and grassland savannas are more susceptible to bush burning and about 75% of the region's crop planting takes place in these zones.

In West Africa, high population areas are concentrated in urban areas, usually the capital or commercial cities with few industries.^14^ However, lack of organized transportation systems in cities has led to a surge in poorly maintained second-hand buses, cars and motorcycles that emit a large quantity of smoke into the environment. The poor road network also contributes to pockets of traffic buildup and increasing elevated emission levels. A higher percentage of farmers in West Africa live in rural areas where they practice subsistence agriculture. This leads to uncontrolled biomass burning, a potent source of NO_2_ emissions in the region.

### Nitrogen dioxide trend and the implications for regional air quality

Air quality over West Africa is becoming increasingly poor with respect to NO_2_ emissions, along with other air pollutants such as tropospheric ozone (O_3_).^15^ The increasing positive trend over major cities in West Africa is an indication of the deterioration of air quality in the region. The major implication of worsening air quality is the effect of pollution on human health. Exposure to NO_2_ over a longer period of time has been linked with severe health issues such as hypertension, diabetes, and heart and cardiovascular diseases.^16^ These health conditions are particularly grave in elderly patients. High fatality rates due to COVID-19 have been reported among people with pre-existing health conditions. For example, Ogen reported that areas that are susceptible to poor air quality as result of high concentrations of NO_2_ over northern Italy and Madrid recorded high fatalities due to COVID-19.^16^

### Impact of meteorological variables on air quality over West Africa

Meteorological variables such as temperature, wind, humidity, cloud cover, solar radiation and rainfall play a significant role in the transformation of pollutants released into the atmosphere. These variables determine the accumulation or dispersion of pollutants from the point of release to the final state of dry deposition or wet scavenging.^17^ Thus, air quality over a region could degenerate or improve as a result of the prevailing weather. Changes in meteorological variables also determine the stability of the atmosphere.^18^ Atmospheric stability, which principally depends on temperature, is an important factor for ambient air quality. Generally, poor air quality is associated with stable atmosphere and vice versa. Song *et al.* and Bell *et al.* explored the impact of synoptic weather on particulate matter (PM) over China and revealed that the contributions of variation in circulation to the reduction in PM_2.5_ levels over North China between 2013 and 2017 were 64% and 45% in summer and winter, respectively.^9^,^19^ In summer, when atmosphere is generally unstable due to warmer temperature, dispersion of PM increases, leading to improved air quality. However, in winter there is a constraint on dispersion due to relatively stable atmosphere which results in poor air quality. When smoke combines with fog in the presence of sunlight (solar radiation) it forms smog, which reduces air quality over cities, especially during the summer when warm temperatures enhance the formation of photochemical smog.^20^

## Methods

The Aura satellite provides a comprehensive global dataset of atmospheric gaseous pollutants such as NO_2_, O_3_ and SO_2_, which has made space-view assessment of pollution possible. Nitrogen dioxide, in particular, has a very short life span of about one day,^21^ and therefore has the highest column density near emission sources. This assists in the detection and characterization of pollutants using space borne instruments. It has also been reported that NO_2_ vertically integrated profiles are highly correlated with surface observations, making the dataset a suitable alternative over data-sparse locations.^22^ Data captured by the ozone measuring instrument (OMI) onboard the Aura satellite are retrieved using the differential optical absorption spectroscopy (DOAS) technique in the visible region (405–465 nm) of the electromagnetic spectrum.^23^ Corrections to the retrieved data included vertical integration of NO_2_ distribution and weighing with altitude-dependent scattering weighting factors. In addition, the retrieved data are considered valid only when cloud cover is between 0–30%. Ozone measuring instrument NO_2_ data has been validated by several authors either by comparing data with similar satellite sensors,^23^ or by *in situ* data.^24^ The OMI NO_2_ agreement with *in situ* data is within a marginal error of about ±20% and a correlation with other sensors of about 0.918 in winter, which varies slightly in different seasons. The mission, which started in 2004, has provided continuous space measurements of tropospheric gases to date, complementing earlier measurements from the Global Ozone Monitoring Experiment (GOME) [1995–2003] and SCanning Imaging Absorption SpectroMeter for Atmospheric CHartographY (SCIAMACHY) [2002–2012].

### Nitrogen dioxide retrieval

From 2007 onward, NO_2_ data from the Aura satellite have become inaccurate due to a systemic error known as row anomaly. Efforts are being made with the aim of creating “refining” algorithms to correct this error and make the data usable for the scientific community. Boersma *et al.*^25^ documented these algorithms provided by various institutions such as the Belgian Institute of Space Aeronomy (BIRA-IASB Qt) (differential Optical Absorption Spectroscopy (QDOAS) retrieval algorithm), Institute of Environmental Physics - Bremen (IUP) (NLIN retrieval algorithm) Netherlands Meteorological Institute (KNMI) (OMNO2^A^ v2 retrieval algorithm) and Max Planck Institute for Chemistry (MPI-C).^25^ Nitrogen dioxide methods of retrieval engaged by these algorithms are, in principle, similar with slight enhancement. They are essentially based on spectral fitting of absorption cross section corresponding to NO_2_ to the top-of-the-atmosphere reflectance spectrum.^26^ Further processing, such as de-stripping and application of air mass factor to convert the NO_2_ residual tropospheric slant column density to tropospheric vertical column density were also carried out. An improvement to these algorithms which takes atmospheric liquid water absorption and intensity correction into consideration to obtain a high quality NO_2_ dataset was initiated and implemented by the European Union (EU) seventh framework (FP7) under the Quality Assurance for Essential Climate Variables (QA4ECV) project.^24^ The QA4ECV NO_2_ dataset made available a complete and consistent set of satellite measurements over a longer period (22 years) from 1995 to 2017 and has been validated over a polluted location in China and yielded a small (~2%) bias. The present study utilizes monthly OMI-NO_2_ data processed by QA4ECV for the period of 2004 to 2017 publicly available from the QA4ECV website.^25^

### Meteorological dataset source

Meteorological variables are an important factor in the determination of air quality over any location. Meteorological data used in this study were obtained from different sources across available global data archives. Air temperature at 2 meters, surface wind speeds and soil moisture were obtained from the European Centre for Medium-range Weather Forecast (ECMWF) fifth generation of atmospheric Re-Analysis (ERA5) dataset, the latest version of the ECMWF re-analysis dataset,^27^ while precipitation data were obtained from the more robust Global Precipitation Climatology Project (GPCP). Other data (vertically integrated moisture flux and convective available potential energy (CAPE)) were obtained from the ECMWF ERA interim dataset. The data sources provide global datasets that have been validated by several authors. For example, GPCP datasets are a merged precipitation product that combines observations and satellite precipitation data to provide a gridded global coverage of precipitation dataset.^28^,^29^ The dataset includes inputs from the special sensor microwave/imager (SSM/I) emission and scattering data, Geostationary Operational Environmental Satellites, Standardized Precipitation Index, outgoing longwave radiation precipitation index data, Television and Infrared Observation Satellite, operational vertical sounder, and Atmospheric Infrared Sounder data and rain gauge observations.^30^,^31^ Both ERA-interim and ERA5 reanalysis datasets are produced by the ECMWF; they have high resolution strength.^24^ The ERA5 is an improvement to the ERA-interim in terms of temporal resolution and data assimilation.^32^ The ERA5 dataset has added ensemble data which was not available in the ERA-interim.^33^ We have made use of vertically integrated moisture flux and CAPE data from the ERA-interim because they are not yet available in the ERA5.

### Nitrogen dioxide statistical and trend analysis

A coefficient of correlation extracts the relationship between two variables and shows the direction of variation between the variables. The Pearson coefficient of correlation (r) given in Equation 2 has been used to determine the influence of some weather variables (precipitation, wind speed, soil moisture and air temperature), on production or otherwise of NO_2_. The r indicator varies from −1 to 1, where a value of +1 is total positive (both variables are increasing or decreasing together), linear correlation, 0 is no linear correlation (both variables do not vary together), and −1 is total negative linear correlation (one the variables is decreasing while other is increasing). Values can range from −1 to +1. The greater the absolute value of the correlation coefficient, the stronger the relationship. The extreme values of −1 and 1 indicate a perfectly linear relationship where a change in one variable is accompanied by a perfectly consistent change in the other where and are any two variables.


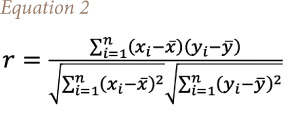


Furthermore, the Mann-Kendall statistic was used to determine the trend in NO_2_ over some selected locations that are prone to poor air quality due to their location, or status as capital cities or commercial hubs in the region. The Mann-Kendall statistic is a non-parametric tool for detecting monotonic trends in a series of environmental, climatic or hydrological data.^34^ Using the statistic, a null hypothesis H_o_ postulates that the data comes from a population with independent realizations and are identically distributed. The alternate hypothesis, H_A_, is that the data follow a monotonic trend. The Mann-Kendall test statistic is calculated according to Equations 3–6:

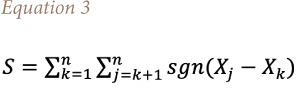
where

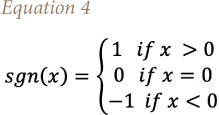
the mean of *S* is *E[S]*=0 and the variance σ^2^ is

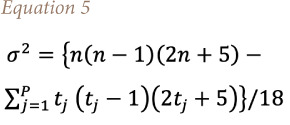
where p is the number of the tied groups in the data set and *t_j_* is the number of data points in the jth tied group. The statistic *S* is approximately normal distributed provided that the following Z-transformation is employed:

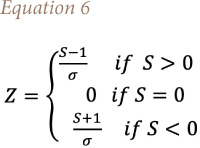



## Results

**Figure 2:**
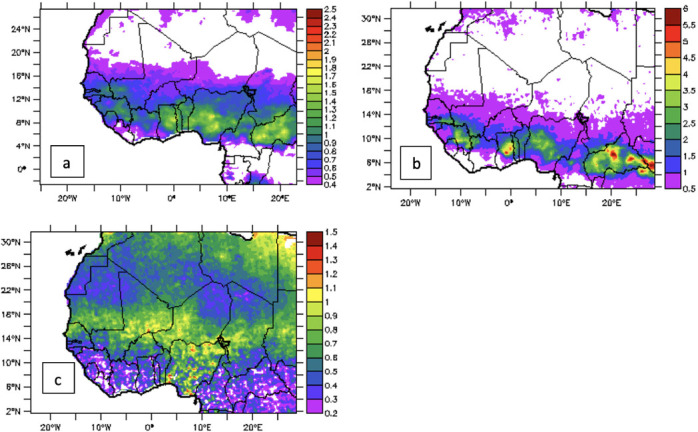
(a) Annual mean value of total nitrogen dioxide (TNO_2_) (b) during the dry season (December, January, February) (c) during the wet season (June, July, August)

The mean annual and seasonal long-term (2004–2017) climatology variation of NO_2_ is presented in Figure 2. The mean annual position over West Africa is between latitude 7°N and 16°N, with the highest concentration band situated over a narrow strip between latitude 8°N and 12°N. The highest concentration (>1.5×10^15^ molec/cm^2^) was found over the central part of Nigeria, Ghana, Togo and Benin. The latitudinal belt with the highest NO_2_ concentration over the region was identified as the savannah woodland, an area that is characterized by scattered trees and abundant tall grasses. The vegetation in this area is controlled by the annual cycle of burning during the dry season and regeneration during the wet season. In the dry months of December, January, February, NO_2_ concentrations are confined to an area between latitude 7°N and 16°N with the core of the highest concentration situated around latitude 10°N and 14°N. This band is partly within the forest zone and woodland zones of the region which also corresponds to the location of the highest long term annual mean NO_2_ concentration. However, the concentration during December, January, February is far higher than the annual mean and the core of the highest concentration is shifted slightly to the south by approximately 1° latitude. The NO_2_ distribution during the months of June, July, August is remarkably different from both the dry and long-term mean values in that the core of highest concentrations is shifted northward between latitude 14°N and 18°N. Concentrations of NO_2_ during June, July, August (maximum of about 2.2×10^15^ molec/cm^2^) are about 50% of December, January, February values. It can be deduced therefore that the majority of NO_2_ pollution is emitted during the dry season when favorable conditions are present.

**Figure 3:**
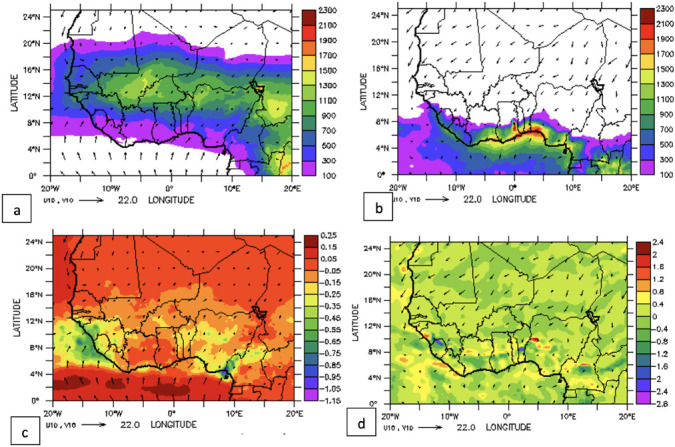
Convective available potential energy (CAPE) JKg^−1^ overlay with wind vector during (a) June, July, August (b) December, January, February. Vertically integrated moisture divergence flux (Kgm^−2^) overlay with wind vector during (c) June, July, August (d) December, January, February.

In Figure 3, the two wind regimes are indicated by wind vectors overlain on convective available potential energy (CAPE) and integrated moisture divergence for both the dry and wet periods. The southwesterly portion of the wind flow is characteristically weak during the dry season and only predominant over the ocean and part of the coastal area in contrast to northeasterly flows which are strong and occupy greater space over the region. The surface meeting point of these wind systems is an area of discontinuity where the vorticity is equal to zero. The two winds are the sweeping factors that gather emissions from sources and accumulate it over the area of discontinuity. Therefore, the location of the point of discontinuity determines the band of the highest NO_2_ concentrations. During the wet period, the band of highest NO_2_ concentrations shifts northward because northbound southwesterly winds are stronger due to increasing trailing moisture. Located about 500 km to the south of the area of discontinuity is a zone of active weather indicated by a high CAPE zone at the rear of the discontinuity area during both the dry and wet periods.^35^ Vertical moisture divergence shown in Figure 3c indicates the moistening of the land surface and boundary layer for moisture build-up and cloud development. The dry period shows patches of moisture flux divergence over the forest zone, however there is widespread moisture divergence during the wet season.

**Figure 4:**
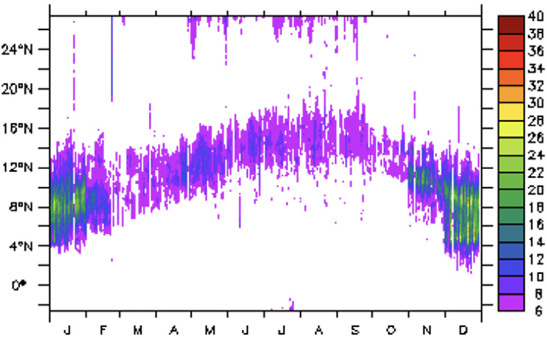
Mean Time–Latitude variation of nitrogen dioxide (NO_2_) [10^14^ molec/cm^2^] averaged between 2011 and 2017

The relationship between moisture flux and NO_2_ concentration is further explored in the next section using the correlation coefficient between soil moisture and NO_2_ pollution level. The latitudinal shift of the heavily polluted band is shown with the aid of a Hovmöller diagram in Figure 4. The south-most position of the core is around 4°N in December, January, February while the north-most position is about 16°N in June, July, August

### Relationships between nitrogen dioxide concentrations and some atmospheric variables

**Figure 5:**
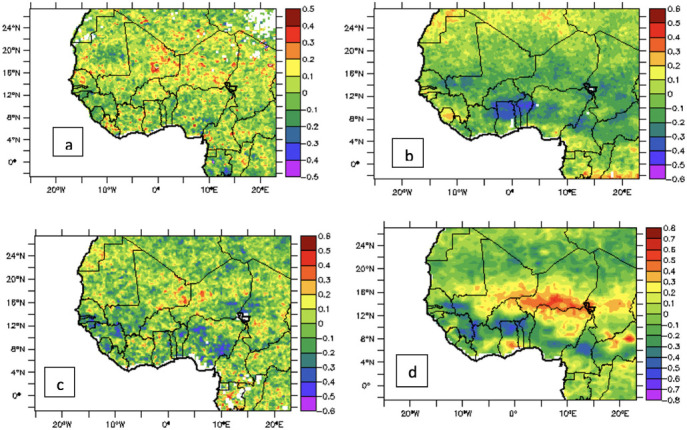
Correlation between total nitrogen dioxide (TNO_2_) and Precipitation (mm) (b) TNO_2_ and surface wind (m/s) (c) TNO_2_ and soil moisture (cm^3^ water per cm^3^ of soil) (d) TNO_2_ and 2 m air temperature (K)

Some environmental factors affect or enhance the production or reduction of NO_2_ such as wind, soil moisture, temperature, and rainfall, etc. The relationship between these factors and NO_2_ was quantified by the coefficient of correlation (r), and the results are presented in Figure 5a (NO_2_ and precipitation), Figure 5b (NO_2_ and surface wind speed), Figure 5c (NO_2_ and soil moisture) and Figure 5d (NO_2_ and air temperature). The coefficient of correlation between NO_2_ and precipitation ranged from −0.5 to +0.5 over different locations of the region and during different seasons. Much of the precipitation which is accompanied by lightning strikes occurs during the wet season and is known to enhance NO_2_ production especially in the upper troposphere.^5^ Consequently, during the wet season more upper tropospheric NO_2_ is produced. During the wet season the relationship between NO_2_ and precipitation is positive and could reach about +0.5 in some locations. In the northern part of the region, thunderstorms embedded in tall clouds account for over 80% of precipitation, consequently NO_2_ has a good correlation with precipitation in that part of the region. During the dry season, lightning as a source of NO_2_ is completely absent and the r between NO_2_ and precipitation is reduced to −0.5 over some locations. The correlation coefficient between NO_2_ and surface wind shows negative values (up to −0.6) over the central part of the region. The central region has been shown to have very high NO_2_ concentrations due to near zero wind speeds, therefore decreasing wind speeds enable accumulation of the pollutant.

Soil moisture also showed a good correlation coefficient with NO_2_ concentration. Over the central part of the region, decreasing soil moisture enhances NO_2_ production. Therefore, increasing soil moisture results in elevated NO_2_ concentrations during the wet season; accordingly, NO_2_ concentrations showed a positive correlation over the northern part of the region. The coefficient of correlation between air temperature and NO_2_ concentrations also show a bipolar signal. Over the central part, high temperature favors the production of NO_2_, resulting in a positive correlation. Negative correlations are also observed over the southern part of the region. It was observed that daytime air temperature (annual average of 35°C) in the central and northern parts of the region is higher by a few degrees than the south (annual average 32°C).

### Linear trend of nitrogen dioxide over selected cities

The trend of NO_2_ over some of these major cities is examined and presented in Figure 6(a–e). The trend over the cities showed a period of peak (high) concentrations alternating with a period of low concentration. The peak periods occur when sources of production of NO_2_ are actively strong with favorable weather conditions for pollution accumulation. Of the five cities considered, Lagos, a coastal city, exhibits the highest concentration during the peak periods. Accra, another coastal and commercial city similar to Lagos showed a high value of NO_2_ during the peak period. However, unlike Lagos, Accra showed evidence of reduced values during the peak period from 2009 onward. As noted in a previous figure (*Figure 2*), the area cutting across the south of Ghana and extending to Liberia is less polluted during both wet and dry seasons.

**Figure 6a:**
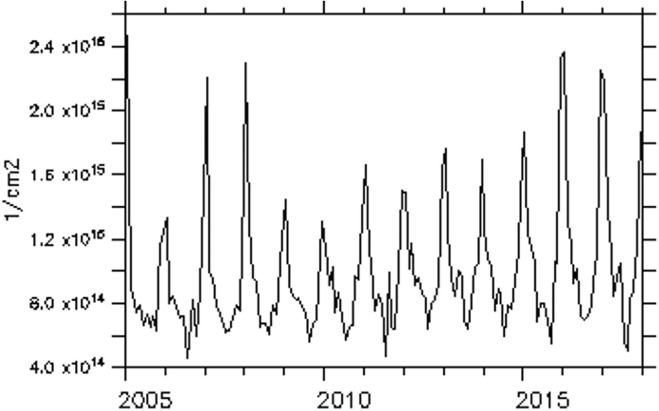
Trend of monthly average of total nitrogen dioxide (TNO_2_) over Lagos

**Figure 6b:**
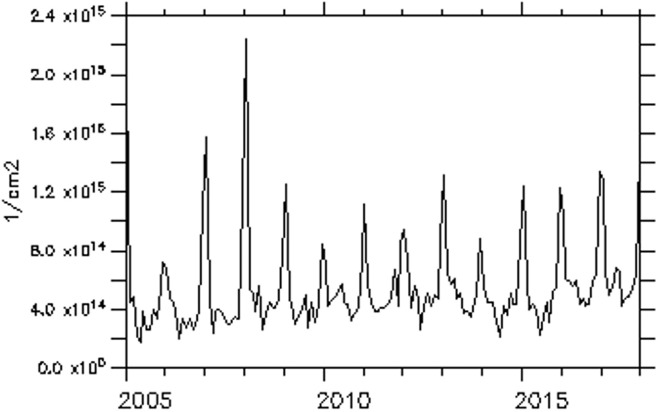
Trend of monthly average of total nitrogen dioxide (TNO_2_) over Accra

**Figure 6c:**
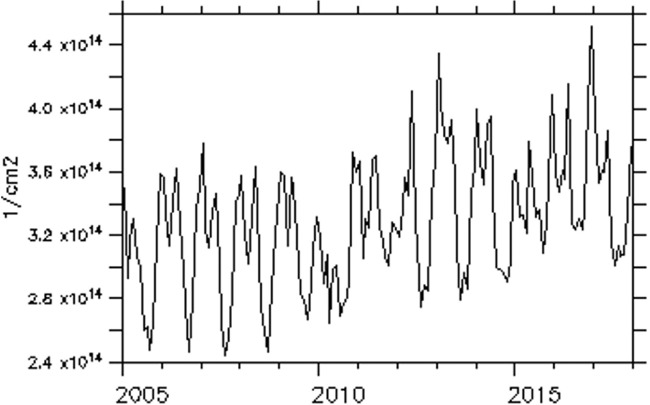
Trend of monthly average of total nitrogen dioxide (TNO_2_) over Bamako

**Figure 6d:**
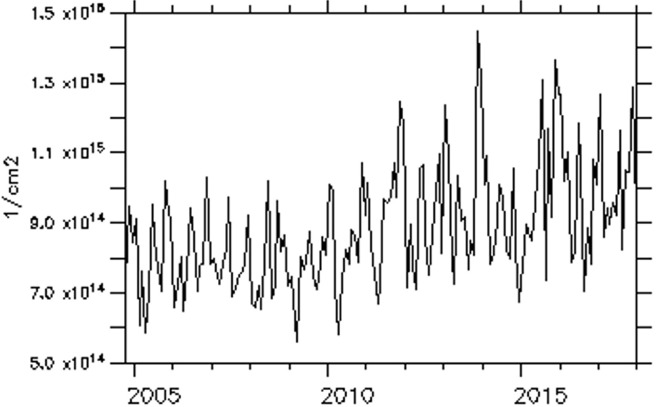
Trend of monthly average of total nitrogen dioxide (TNO_2_) over Dakar

**Figure 6e:**
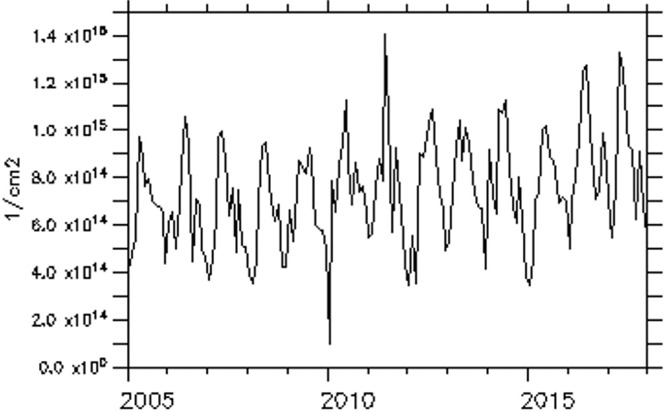
Trend of monthly average of total nitrogen dioxide (TNO_2_) over Niamey

Over Bamako, Dakar and Niamey, the NO_2_ trend showed an increasing value during the peak period. The trend equations of NO_2_ over the selected cities are shown in Table 1a. The trend equation revealed a growing rate of NO_2_ pollution over the cities, with the fastest rate found in Dakar. The results of the Mann-Kendall statistic as shown in Table 1b also revealed an increasing upward trend of NO_2_ in all of these cities with statistically significant trends at a p-value of less than α= 0.05. In addition, Lagos had the highest monthly average maximum values, while Niamey had the lowest monthly average value of 9.53×10^13^ molec/m^2^.

**Table 1a:** Linear Trend of Characteristics of Tropospheric Column total nitrogen dioxide (TNO_2_) for Selected Cities in West Africa

**City**	**Linear Trend equation**	**Monthly Maximum**	**Monthly Minimum**
Lagos	Y=5×10^10^×−10^15^	2.46×10^15^	4.64×10^14^
Accra	Y=3×10^10^×−6×10^15^	2.25×10^15^	1.80×10^14^
Bamako	Y=1×10^10^×−10^14^	4.52×10^15^	2.44×10^14^
Dakar	Y=6×10^10^×−10^15^	1.45×10^15^	5.59×10^14^
Niamey	Y=5×10^10^×−10^15^	1.41×10^15^	9.53×10^13^

**Table 1b:** Mann-Kendall statistic of total nitrogen dioxide (TNO_2_) Trend for Selected Cities in West Africa

**City**	**Mann Kendall statistic (α=0.05. n=156)**				
	S	Variance (*σ*^2^)	Z-stat	P-value	Trend
Lagos	1923	28120.09825	0.06834969	0.002868273	Increasing
Accra	2871	51291.08531	0.055955143	0.003321340	Increasing
Bamako	2841	50489.3917	0.05624944	0.002134321	Increasing
Dakar	3799	78067.15764	0.04865042	0.003600123	Increasing
Niamey	2226	35019.71838	0.063535634	0.004000134	Increasing

Abbreviation: S, Mann-Kendall statistic

### Elevated NO_2_ level and COVID-19

In West Africa, as shown in Table 2, the highly populated and polluted area of Lagos, Nigeria experienced a high fatality rate due to COVID-19. Although a one-on-one relationship between COVID-19 and NO_2_ pollution is not yet established from any reported studies, recent studies, however, have shown that regions of the world that are heavily polluted recorded high fatality rates for COVID-19.^36^ As the trends of NO_2_ increased over major cities in West Africa, the population in these cities is vulnerable to health hazards caused by air pollution which may result into high cases of air pollution-related diseases capable of weakening the immune system (*Figure 6*).^37^ A population with a low immune system is highly vulnerable to mortality due to pandemics such as COVID-19. This was demonstrated in the case of northern Italy where pre-existing health conditions contributed to high fatality rates for COVID-19.^36^,^38^,^39^

**Table 2:** COVID-19 fatalities as of November 2020 for Selected Cities in West Africa^45^

**City**	**Population (million)**	**Number of fatalities**
Lagos	14.4	208
Accra	2.5	168
Bamako	2.6	125
Dakar	2.5	152
Niamey	1.3	69

## Discussion

In West Africa, the impact of increasing population on the region's air quality is still not well researched due to the lack of data measurements and therefore, sources of pollution are not well regulated. This has raised the level of air pollution over the region. Nitrogen dioxide as a major air quality criteria pollutant was examined using data available from satellite measurements. It should be noted that satellite measurements have both advantages and limitations as a means of measuring ground level pollution. The major advantage of satellite measurements includes large area data coverage and continuous measurements, among others. Limitations of the measurements are that they exhibit biases due to algorithms designed for data retrieval and missing data because of swath coverage. In West Africa, the lack of measurements from ground-based instruments has created a wide research gap in the field of air quality monitoring. The results from this study considered the pollution episode due NO_2_ over the region. Nitrogen dioxide concentrations are higher during the dry period not only because there are higher emissions, but because there are suppressions of cloud growth which prevent upper atmospheric layer venting. During the wet period, the development of tall clouds over an area at the rear of the discontinuity zone ensures sufficient venting of NO_2_ vertical column density which results in a significant reduction of NO_2_ concentrations. It is also worth noting that an exceptional increase of NO_2_ over the region is strongly linked with the long-range transport from central Africa to the region.^40^ With the aid of strong easterly winds, most of the pollutants are transported from the densely forested area of the basin during the period of bush burning.

Dry periods are characterized by widespread bush burning which releases large quantities of emissions into the atmosphere. In addition to bush burning, there are other sources of NO_2_ emissions in the West African region such as vehicular emissions in large cities, household generators, industrial power plants and emissions from vegetation due to microbial activities. These sources are active throughout the year, however with varying intensities. Of all of these emission sources, only bush burning shifts with latitude during the course of a year. The shift is as a result of the migration of rain producing system and cropping season over the region. Preparations for cropping season first begin in the southern rain forest zone during the months of DJF, resulting in enhanced active bush fires. Cropping season later shifts to the north in the months of June and July, also accompanied with active bush fire. Although cropping and bush fire shift with latitude over the region, the two wind systems (southwesterly and northeasterly) play a more important role in the formation of bands of high NO_2_ concentrations. The seasonal pattern shown in the trend of NO_2_ is specifically due to large-scale circulations which modulate the convergence and accumulation of pollution. The implication of this is that local pollution estimation by satellite is very poor. However, the large-scale signal is indicative of predominant activities that are responsible for pollutant generation. During the peak of the pollution episode in the dry season, bush burning and forest wildfires are the most potent sources of NO_2_. During the wet season, the peak of NO_2_ can be linked to large scale atmospheric transport of NO_2_ from other active regions, especially the central Africa region. The hotspot of pollution in West Africa, on a large scale, is dictated by the location of ITD. Therefore, places or areas where ITD is located will have almost stagnant circulation, poor pollution ventilation and dispersion due to near zero vorticity in the vicinity of ITD. This stagnation results in very poor air quality due to pollution accumulation. Since large cities are prone to large emissions from industries and increasing vehicular volume, a period when ITD is stagnant over them marks the peak of poor air quality and increasing hospitalization for pollution-related diseases. Meteorological variables such temperature, wind speed, soil moisture etc. also play important role in NO_2_ pollution episode. They could either act to elevate the pollution level or alleviate it as indicated from the results of this study. For example, decreasing moisture reduces the greenness of vegetation, making them vulnerable to wildfire during the dry season. In a similar study limited to Nigeria only, Peel *et al.* reported that stagnant winds, at the point of discontinuity, contribute significantly to seasonal atmospheric pollution of the region.^2^ Unlike in the eastern United States, parts of Europe and Asia where the major sources of NO_2_ emission are industrial power generation plants and vehicular emissions and where the areas of NO_2_ heavy pollution do not shift seasonally,^41^ over west Africa, there is a seasonal shift of hot spots as a result of south-north migration of monsoon winds. However, during the wet season, increasing soil moisture causes vegetation to blossom leading to production of NO_2_ as a result of microbial activities in the root of crops. Similarly air temperature also significantly affects NO_2_ pollution episodes. However, the difference in temperature between the north and the south may not be the cause of observed differences in the correlation coefficient between the north and south; rather the difference is attributable to the effects of fertilizer application. The northern and central parts of the region's agricultural activities are heavily supported by fertilizer application. The fertilized soil could emit more NO_2_ under high temperatures, a fact that has been demonstrated by researchers such as Oikawa *et al.*, who found that a fertilized soil could release as much as 0.46 gN-NOx m^−3^ compared to 0.037 gN-NOx m^−3^ for non-fertilized soil.^42^ Along the coastal zone, there is a high coefficient of correlation between air temperature and NO_2_ which is attributable to emissions from vehicles and oil and gas activities, since most of the region's commercial and capital cities which experience a high traffic volume and industrial activities are located along the coast. Major and capital cities are prone to a high level of pollution due to emissions from transportation, domestic cooking, burning of refuse, etc. Therefore, the strong NO_2_ pollution signal over Lagos is a consequence of high population density, high traffic volume and petrochemical activities, among others. Similarly, two major factors could be responsible for reduced NO_2_ observations over Accra; unfavorable climatic conditions for pollution accumulation or efficient control of production factors such as reduction in burning or traffic emissions. Adedokun observed a characteristic strong influence of the descending branch of Walker circulation over Accra, which gives Accra and neighboring areas unusually dry weather compared to similar coastal locations.^43^ The descending Walker circulation inhibits cloud growth, enhances divergence at the surface and therefore produces less rain. Thus, it can be inferred that the same weather condition is responsible for de-accumulation of pollution over the area. Poor quality of air at a particular location over a long time, weakened immunity increases susceptibility to various diseases. The COVID-19 pandemic has demonstrated that cities with poor air quality experienced higher fatalities as a result of COVID-19, as in Italy and Spain.^44^ In West Africa, Lagos, Nigeria, falls into the category of heavily polluted cities with high fatalities due to COVID-19.

### Limitations of this study

Non-availability of ground station data made actual comparison of satellite pollution emissions data with in-situ data difficult over West Africa. It is therefore possible that emissions from certain locations may be underestimated or overestimated due to inaccurate satellite retrieval algorithms and/or unaccounted local sources of pollution.

## Conclusions

Emissions of certain air pollutants such as NO_2_ are a major determinant of air quality in a particular location. The present study examined the spatio-temporal spread of NO_2_ concentrations in West Africa for both the wet and dry seasons. The highest annual concentration of NO_2_ is found in the vicinity of the inter-tropical discontinuity zone, where wind speeds are typically low and horizontal vorticity is zero. The source of NO_2_ strength over the region varies with season and local emissions. Typical sources of emissions are mainly agricultural, vehicular, and industrial emissions and bush burning. In the present study, the movement of the ITD line from south to north during the transition period from dry to the wet season corresponds to the south-north shift of the region's air pollution hot spots. The worst episode of pollution in the region occurs during the dry season when bush burning is at the peak and ITD temporarily become stationary over the southern part. This period also coincides with the peak of hospitalization for air pollution-related diseases such as cough and sore throat. The temporal trend of NO_2_ in some selected cities showed increasing pollution between 2004 and 2017 (study period). This increasing trend may lead to increasing air pollution-related hazards, deteriorating human health and impact immunity of the population living in hot spot cities. The implications of increasing pollution were discussed in relation to human health. Areas prone to elevated air pollution were found to have a record of high COVID-19 fatalities due to weakened immunity which indicated that the increasing trend of pollution over West Africa poses a risk to public health and increases vulnerability to mortality due to pandemics such as COVID-19.
